# The consequences of delaying insulin initiation in UK type 2 diabetes patients failing oral hyperglycaemic agents: a modelling study

**DOI:** 10.1186/1472-6823-9-19

**Published:** 2009-10-05

**Authors:** Gordon Goodall, Eric M Sarpong, Clarice Hayes, William J Valentine

**Affiliations:** 1 IMS Health, Allschwil, Switzerland; 2 Global Health Outcomes, Eli Lilly & Co., Indianapolis, USA; 3 Ossian Health Economics and Communications, Basel, Switzerland

## Abstract

**Background:**

Recent data have shown that type 2 diabetes patients in the UK delay initiating insulin on average for over 11 years after first being prescribed an oral medication. Using a published computer simulation model of diabetes we used UK-specific data to estimate the clinical consequences of immediately initiating insulin versus delaying initiation for periods in line with published estimates.

**Methods:**

In the base case scenario simulated patients, with characteristics based on published UK data, were modelled as either initiating insulin immediately or delaying for 8 years. Clinical outcomes in terms of both life expectancy and quality-adjusted life expectancy and also diabetes-related complications (cumulative incidence and time to onset) were projected over a 35 year time horizon. Treatment effects associated with insulin use were taken from published studies and sensitivity analyses were performed around time to initiation of insulin, insulin efficacies and hypoglycaemia utilities.

**Results:**

For patients immediately initiating insulin there were increases in (undiscounted) life expectancy of 0.61 years and quality-adjusted life expectancy of 0.34 quality-adjusted life years versus delaying initiation for 8 years. There were also substantial reductions in cumulative incidence and time to onset of all diabetes-related complications with immediate versus delayed insulin initiation. Sensitivity analyses showed that a reduced delay in insulin initiation or change in insulin efficacy still demonstrated clinical benefits for immediate versus delayed initiation.

**Conclusion:**

UK type 2 diabetes patients are at increased risk of a large number of diabetes-related complications due to an unnecessary delay in insulin initiation. Despite clear guidelines recommending tight glycaemic control this failure to begin insulin therapy promptly is likely to result in needlessly reduced life expectancy and compromised quality of life.

## Background

The global impact of type 2 diabetes on patients and healthcare systems is difficult to overstate given the sheer numbers of individuals with, and the chronic and pervasive nature of, the condition. By 2025 it is estimated that 333 million adults will suffer from diabetes [1] and 90% of these will have type 2 diabetes. Economic consequences for healthcare payers are similarly dramatic with the most recent cost estimates in the US alone at $116 billion for direct costs (pharmacy, hospitalisation, etc) and a further $58 billion due to lost productivity as a direct consequence of complications arising from the disease [2]. There have been similar findings in Europe where the Cost of Diabetes in Europe (CODE-2) study estimated total annual expenditure for type 2 diabetes in eight countries [3]. Notably both studies highlighted the contribution of hospitalisation costs as a key driver (responsible for at least 50% of the total value) [2,4] and the fact that the costs of treating patients with diabetes are higher than for those without [2,5]. In the UK alone, it is estimated that there are over 2.2 million individuals with diabetes and 5% of the total national healthcare budget (over £1 billion annually) is spent on treatment and management of the condition [6].

When managing type 2 diabetes the general focus is on achieving and maintaining good glycaemic control while minimising the potential for adverse events such as hypoglycaemia. The value of this approach has been shown from evidence gained in landmark clinical and epidemiological studies where the reduced incidence of micro- and macrovascular complications was apparent with intensive glycaemic control [7-14] and has further been confirmed in a published meta-analysis of observational studies [15]. To achieve these reductions in the incidence of complications, current UK guidelines from the National Institute for Health and Clinical Excellence (NICE) recommend target glycosylated haemoglobin (HbA1c) levels of between 6.5 and 7.5% (targets that are in line with guidance from other national and international bodies) [16]. Although this dogma has come under scrutiny and been challenged by some studies [17] it remains the central principal for the majority of treatment regimens [18] even if the initiation of insulin is often resisted by both patients and physicians [19].

Despite published guidance on the management of diabetes it is apparent that many patients fail to receive the appropriate standard of care (although the reasons are unclear and blame cannot be easily apportioned). The magnitude of this issue has been demonstrated by two studies examining type 2 diabetes management in the UK primary care setting [20,21]. In these retrospective cohort studies the authors found that not only were HbA1c measurements poorly recorded for the majority of patients, regardless of numbers of oral antidiabetic agents (OADs) prescribed, but even where data were available at least 40% of individuals failed to achieve a modest HbA1c target of 7.5% [21]. Despite this poor control the average time spent on monotherapy was 3.8 years and even after failing to achieve glycaemic control with two or more OADs the median time before commencing insulin therapy was 7.7 years from initiation of the final OAD [20,21]. This implies an average time to initiation of insulin of at least 11.5 years from initial diagnosis of type 2 diabetes. From the published data it is clear then that patients would not be maintaining their glycaemic control targets for a substantial proportion of this time. This has the obvious implication that patients are unnecessarily at an elevated risk of diabetes-related complications.

There are several interesting observations relating to the management of type 2 diabetes to come from these studies including: the infrequent measurement of HbA1c despite clear guidelines that this is the preferred measure of glycaemic control, the potential therapeutic benefits of multiple OADs treatment regimens, and the apparent reluctance of doctor or patient to initiate insulin. We have chosen to focus this investigation on the seemingly protracted delay to the initiation of insulin that UK patients are exposed to and it's potential consequences. Using a computer simulation model of long-term type 2 diabetes progression and based on published data the aim of this analysis was to compare the difference in projected lifetime clinical outcomes for patients immediately initiating versus delaying initiation of insulin.

## Methods

### Model

A brief overview of the CORE Diabetes Model is provided here, but a full description of the model has been previously published by Palmer *et al*. [22,23]. The model is a non-product-specific diabetes policy analysis tool that takes into account intensive or conventional insulin therapy, oral hypoglycaemic medications, screening and treatment strategies for micro-vascular complications, treatment strategies for end-stage complications and multi-factorial interventions. Disease progression is based on a series of inter-dependent sub-models that simulate progression of disease-related complications (angina, myocardial infarction, congestive heart failure, stroke, peripheral vascular disease, diabetic retinopathy, macula oedema, cataract, hypoglycaemia, ketoacidosis, lactic acidosis, nephropathy, end-stage renal disease, neuropathy, foot ulcer and amputation) as well as mortality from other causes. Each sub-model uses time, state and diabetes type-dependent probabilities derived from published sources. The reliability of simulated outcomes has been tested, with results validated against those reported by clinical trials and epidemiological studies [23].

The model reports outcomes in terms of life expectancy (years), quality-adjusted life expectancy (quality-adjusted life years), cumulative incidence of complications and time free of complications. Quality of life outcomes are calculated using diabetes-specific state and event utilities from published studies [24-27].

### Hypothetical Treatment Progressions and Modelling Analyses

The simulated treatment and patient inputs were based on reported data from two recent retrospective cohort studies of type 2 diabetes management in the UK [20,21]. In the base case analysis hypothetical UK patients were assigned to either insulin initiation in year 1 of the simulation, mimicking the administration of insulin following failure of OADs to maintain glycaemic control, or after 8 years, simulating the observed length of delay in line with published data [20]. Insulin efficacy in terms of effect on HbA1c levels, weight change and hypoglycaemia event rates were taken from a recent randomised clinical trial [28] (Table 1) as they reflect the observed change in HbA1c levels reported in the cohort study [20]. In order to simplify the analyses no change in other parameters (e.g. systolic blood pressure, serum lipids, etc.) were assumed for either treatment. In line with published evidence from the UKPDS HbA1c was assumed to increase by 0.15% in all years [9]. Simulations were performed over a 35 year time horizon to ensure all outcomes were accounted.

**Table 1:** Treatment effects associated with insulin initiation

**Scenario**	**HbA1c change (%)**	**BMI change (kg/m^2^)**	**Hypoglycaemia event rate (per 100 patient years)**	**Reference**
Base case	-1.30	+1.53	570	[28]
Higher HbA1c	-1.98	+3.70	310	[29]
Lower HbA1c	-0.80	+1.90	230	[28]

HbA1c -- glycosylated haemoglobin; BMI -- body mass index

### Simulation Cohort and Reported Outcomes

Patient characteristics were taken from published studies in the UK and are detailed in Table 2. Undiscounted life expectancy (years), quality-adjusted life expectancy (quality-adjusted life years), cumulative incidence of complications and time free of complications were calculated for each simulation and are reported where appropriate. Full results of all simulations including all outputs are available on request from the authors.

**Table 2:** Simulation cohort baseline characteristics

**Characteristic**	**Value**	**Reference**
Age (years)	64.2	[20]
Duration of diabetes (years)	4	[21]
Percentage male (%)	55.26	[20]

*Clinical parameters*		
HbA1c (%)	8.16	[20]
SBP (mmHg)	141	[30]
Total cholesterol (mg/dl)	214.5	[30]
HDL-cholesterol (mg/dl)	42.9	[30]
LDL-cholesterol (mg/dl)	128.7	[30]
Triglycerides (mg/dl)	204.7	[30]
BMI (kg/m^2^)	30.1	[20]

*Ethnicity*		
Proportion White (%)	82	Assumed*
Proportion Black (%)	9	Assumed*
Proportion Asian (%)	9	Assumed*

*Comorbidities at baseline*		
Myocardial infarction (%)	8.2	[31]
Peripheral vascular disease (%)	4.5	[30]
Stroke (%)	6.6	[30]
Congestive heart failure (%)	22.8	[30]
Microalbuminuria (%)	0.7	[31]
End-stage renal disease (%)	1	[31]
Background diabetic retinopathy (%)	17.7	[31]
Neuropathy (%)	6.5	[31]

HbA1c -- glycosylated haemoglobin; SBP -- systolic blood pressure; HDL -- high-density lipoprotein; LDL -- low-density lipoprotein; BMI -- body mass index; * no data found and so assumed

### Statistical Methodology and One-Way Sensitivity Analyses

For each simulation performed a cohort of 1,000 patients was run through the model 1,000 times to address first-order uncertainty (random variation due to chance events). No statistical uncertainty around patient or treatment parameters was investigated due to the hypothetical nature of the analysis, however, one-way sensitivity analyses were performed to investigate the effects of assumptions on outcomes. In these scenarios individual aspects of the simulations were varied where: time to initiation of insulin was varied between 4 and 8 years; clinical benefits associated with insulin initiation from two alternative studies were accounted [28,29]; and no disutilities were accounted for hypoglycaemia events to highlight the effect of this on quality of life.

## Results

### Main Findings

**Figure 1:**
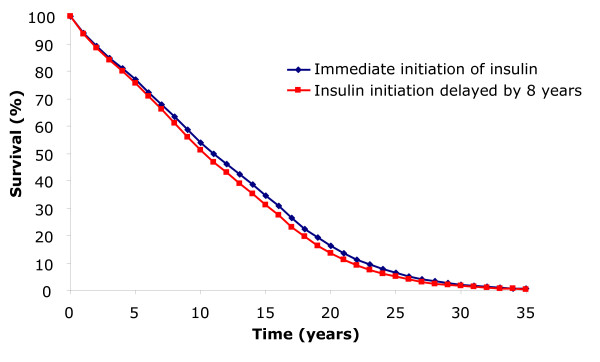
**Survival curve for simulated cohort initiating insulin immediately versus delaying for 8 years**.

When initiating insulin immediately, life expectancy was projected to increase by an average of 0.61 years (≈7.5 months) in comparison with a delay to insulin initiation of 8 years (11.40 versus 10.78 years) (Table 3). Survival curves show an obvious reduction in mortality after just 4 years of the simulation for those initiating insulin immediately versus delaying initiation, and this remains evident for the majority of the simulation period (Figure 1). There is also an improvement in quality-adjusted life expectancy where a benefit of 0.34 quality-adjusted life years (QALYs) is associated with immediate versus delayed initiation of insulin (7.53 versus 7.19 QALYs) (Table 3). This benefit is projected despite the additional hypoglycaemia risk individuals who immediately initiated insulin would experience in the first 8 years of the simulation versus those with delayed initiation (570 events per 100 patient years). Both of these clinical outcomes are driven by substantial reductions in the cumulative incidence and time to onset of most diabetes-related complications due to the improvements in glycaemic control associated with insulin administration (Table 4, Table 5).

**Table 3:** Results of immediate initiation of insulin versus 8 year delay

	**Immediate Initiation**	**8-year Delay**
**Life expectancy (years)**	11.40 ± 0.25	10.78 ± 0.24
***difference***	0.61	
**Quality-adjusted life expectancy (QALYs)**	7.53 ± 0.17	7.19 ± 0.16
***difference***	0.34	

QALY -- quality-adjusted life year; discrepancies are due to rounding

**Table 4:** Cumulative incidence of diabetes-related complications

	**Immediate initiation**	**8-year delay**	**Difference**
**Complication**			
Congestive heart failure, onset (%)	29.22	30.08	-0.87
Congestive heart failure, death (%)	26.58	26.26	+0.31
Angina (%)	12.85	13.13	-0.27
Myocardial infarction, event (%)	28.98	33.63	-4.65
Myocardial infarction, death (%)	25.05	28.07	-3.02
Stroke, event (%)	8.60	8.49	+0.12
Stroke, death (%)	4.73	4.62	+0.12

Background retinopathy (%)	14.08	17.70	-3.62
Proliferative retinopathy (%)	0.91	1.22	-0.31
Severe vision loss (%)	4.05	5.28	-1.22
Macular oedema (%)	11.73	14.81	-3.09
Cataract (%)	6.77	7.45	-0.68

Microalbuminuria (%)	18.66	23.21	-4.56
Gross proteinuria (%)	3.97	5.17	-1.20
End-stage renal disease (%)	0.59	0.75	-0.16

Foot ulcer, first (%)	18.22	20.09	-1.87
Foot ulcer, recurrence (%)	18.62	20.54	-1.92
First amputation (%)	4.37	4.72	-0.35
Neuropathy, onset (%)	34.88	40.49	-5.61
Peripheral vascular disease, onset (%)	13.80	15.90	-2.10

**Table 5:** Time to onset of diabetes-related complications (years)

**Complication**	**Immediate initiation**	**8-year delay**	**Difference**
Any complications	3.08	2.33	0.75

Congestive heart failure	8.90	8.32	0.58
Angina	10.47	9.87	0.60
Myocardial infarction	10.04	9.28	0.76

Stroke	10.43	9.86	0.57
Background retinopathy	8.48	7.68	0.80
Proliferative retinopathy	11.30	10.67	0.63
Severe vision loss	11.13	10.43	0.70
Macula oedema	10.48	9.57	0.91
Cataract	10.84	10.17	0.67

Microalbuminuria	10.03	9.04	0.99
Gross proteinuria	11.20	10.51	0.69
End-stage renal disease	11.37	10.76	0.61

Foot ulcer	10.44	9.73	0.71
Amputation	11.19	10.57	0.62
Neuropathy	8.16	7.07	1.09
Peripheral vascular disease	10.12	9.36	0.76

### Sensitivity Analyses

In all sensitivity analyses modelled improvements in clinical outcomes for immediate versus delayed initiation of insulin remained (Table 6). There were substantial benefits in life expectancy even when the time to initiation was reduced to 6 or 4 years. This difference in life expectancy for immediate versus delayed insulin initiation fell from 0.61 years in the base case to 0.57 and 0.48 years with a delay of 6 and 4 years, respectively. Interestingly there was little difference in quality-adjusted life expectancy for the same sensitivity analyses where it was reduced from 0.34 QALYs in the base case to 0.33 and 0.28 QALYs for a delay of 6 and 4 years, respectively. This was driven by the reduced effect of hypoglycaemia on quality of life offsetting the difference in incidence of complications.

**Table 6:** Sensitivity analyses results

	**Life expectancy (years)**			**Quality-adjusted life expectancy (QALY)**		
	**Immediate Initiation**	**Delayed Initiation**	**Difference**	**Immediate Initiation**	**Delayed Initiation**	**Difference**
*Base case*	*11.40 ± 0.25*	*10.78 ± 0.24*	*0.61*	*7.53 ± 0.17*	*7.19 ± 0.16*	*0.34*
Initiation of insulin - 6 year delay	11.40 ± 0.25	10.83 ± 0.25	0.57	7.53 ± 0.17	7.20 ± 0.17	0.33
Initiation of insulin - 4 year delay	11.40 ± 0.25	10.91 ± 0.25	0.48	7.53 ± 0.17	7.25 ± 0.17	0.28
Higher HbA1c change	11.66 ± 0.27	10.85 ± 0.23	0.82	7.94 ± 0.18	7.31 ± 0.16	0.63
Lower HbA1c change	11.11 ± 0.24	10.74 ± 0.25	0.37	7.48 ± 0.17	7.23 ± 0.17	0.24
No disutility for hypoglycaemia	11.40 ± 0.25	10.78 ± 0.24	0.61	7.88 ± 0.17	7.34 ± 0.16	0.55

QALY -- quality-adjusted life expectancy; HbA1c -- glycosylated haemoglobin; discrepancies are due to rounding.

Where different treatment effects associated with insulin were applied the magnitude of improvements in clinical outcomes was altered but the overall conclusions were the same. For an immediate versus 8 year delay in the initiation of insulin the difference in life expectancy was 0.82 and 0.37 years and difference in quality-adjusted life expectancy was 0.63 and 0.24 QALYs for higher and lower estimates of HbA1c change, respectively.

If the disutility associated with hypoglycaemic events was eliminated there was an increase in the improvement in quality-adjusted life expectancy of 0.21 QALYs compared to the base case simulation (0.55 versus 0.34 QALYs) for immediate versus delayed initiation of insulin.

## Discussion

Although it may be an unsurprising conclusion to draw that, improving glycaemic control earlier versus later leads to clinical benefits over time, the increase in mean life expectancy of over 7 months projected in this study is quite substantial. We have presented evidence here that in the UK setting, in line with published evidence on the average time spent with poorly or uncontrolled hyperglycaemia prior to initiating insulin, individuals are projected to experience improvements in life expectancy, quality-adjusted life expectancy and a reduced incidence and delayed onset of complications regardless of the delay to initiation and assumed efficacy of insulin.

Even though there is a notable risk of hypoglycaemia which would at least partly offset improvements in quality-adjusted life expectancy associated with insulin administration there were still substantial benefits for the immediate versus delayed initiation of insulin. By performing a sensitivity analysis where no quality of life disutility was accounted for hypoglycaemia events the effect on outcomes of this consequence of improved glycaemic control was evaluated. Overall the incidence of hypoglycaemia associated with insulin therapy led directly to a decrease in quality-adjusted life expectancy of 0.21 QALYs compared to the base case analysis (0.55 versus 0.34 QALYs).

Model outcomes have been presented here as undiscounted values to more clearly demonstrate the potential outcomes for immediate versus delayed initiation of insulin. Although NICE recommends a discount rate of 3.5% *per annum *in the UK setting we have deliberately chosen not to conduct this study from an economic standpoint, but rather, relate only the potential clinical benefits that are foregone by delaying insulin initiation. However, as a matter of record, where outcomes were discounted the results for the base case showed a benefit for immediate versus delayed initiation of insulin in terms of life expectancy of 0.36 years (8.57 versus 8.20 years) and for quality-adjusted life expectancy of 0.18 QALYs (5.73 versus 5.55 QALYs). Although these discounted figures may not appear as dramatic as the undiscounted results they still represent substantial improvements that are in line or better than many health interventions seeking market approval.

As noted, this study makes no attempt to quantify the economic consequences of delaying insulin initiation. However there are likely to be direct medical cost savings associated with immediate initiation of insulin due to diabetes-related complications avoided. There are also other potential cost savings from a societal perspective, e.g. less need for carers, sustained productivity due to premature mortality or retirement avoided, etc. that are not accounted here.

Although the results from this modelling analysis unequivocally project improved outcomes for those initiating insulin and attaining HbA1c targets earlier than currently observed in the UK there are limitations to the study and these must be acknowledged. Most obviously this is a hypothetical modelling study and the outcomes are not based on randomised controlled clinical trial data (which would be ethically impossible to acquire). However, the inputs are based on recent real world data from the UK setting and where possible all data required to generate the analyses have been taken from the primary study publications [20,21] and then supplemented with other UK-specific data [30,31].

Additionally we have attempted to avoid bias by performing the analysis without a product-specific focus and it represents no particular commercial standpoint. In terms of the tool used to perform the analyses, the computer simulation model was developed as, and remains, a non-product-specific decision analysis tool that has been validated against a number of published clinical trials and shown to project robust and plausible outcomes [23]. In order to avoid unnecessary complexity and aid transparency the analysis was designed to allow minimal opportunities for bias or error (most inputs are unchanged between comparators) and was simplified where necessary. For example, treatment effects associated with insulin in terms of changes to systolic blood pressure and serum lipids (total cholesterol, high- and low-density lipoprotein cholesterol and triglycerides) were not modelled. This was intended to both simplify and maintain a conservative approach as improvements in these parameters were likely to reduce cardiovascular outcomes and one would predict that this would favour the immediate versus delayed initiation of insulin scenario.

Another potential criticism of this analysis is that it does not account any insulin specific effects outside of change in HbA1c and risk of hypoglycaemia. Although there is a distinct absence of reliable data regarding safety issues with insulin, one area that merits specific attention is in individuals administering insulin subsequent to an MI event. The published epidemiological data from the DIGAMI-2 trial reported a significant increase in the cumulative endpoint of death, reinfarction and stroke (hazard ratio of 1.42) for those administering insulin subsequent to an MI event [32]. However, these results are in contrast to outcomes from the original DIGAMI study [33] and the two conclusions are challenging to resolve with each other. As the authors of the post-hoc analysis of the DIGAMI-2 trial themselves point out, there is the strong possibility that results may be confounded by unknown covariates, and in a complex disease such as diabetes this is a common concern. As there is a limited amount of data regarding this in the context of the analysis presented here no attempt has been made to account for a potential increase in further cardiovascular events following MI when administering insulin. It should be noted though that in the base case analysis the time to onset for a first MI event was greater than the delay to initiation of insulin and so for the majority of cases there would have been no difference in outcomes (i.e. both arms would have been administering insulin).

Additionally, a similar analysis could be carried out whereby glycaemic control is postulated to be better maintained by any manner (more appropriate prescription of OADs or other pharmaceutical interventions, compliance issues, patient education, physician support, etc.), however it was decided that with the published evidence available the use of insulin represented a more realistic scenario. It should be noted that the publications by Calvert and colleagues which prompted this analysis did not directly assess the make up of the OAD based regimens and whether there were any issue with patient compliance. However, glycaemic control measurements for individuals were often not recorded perhaps indicating a systematic failure to manage the patients condition regardless of the intervention used. Regardless of the specific management practice addressed in this context the results clearly highlight the potential benefits for type 2 diabetes patients in the UK who attain glycaemic control targets compared with the current "real world" observations.

By considering only insulin effects we excluded the possibility that patients may have been prescribed one of the new classes of diabetes interventions (DPP-IV inhibitor or GLP-1 agonist) prior to initiating insulin. As these new compounds reach the market and become available for prescription they may well be used prior to insulin initiation, however, the data available to us largely predated their use and so they were not considered in this analysis. Their use and effect on the glycaemic control of type 2 diabetes subjects in the UK remains to be demonstrated.

We have performed this study using a computer simulation model that incorporates risk formula generated from studies that demonstrated benefits associated with intensive glycaemic control in terms of hard clinical endpoints. Following on from these findings the attainment and maintenance of good glycaemic control as judged by HbA1c measurements is still recommended in most patient groups by national healthcare bodies. It has to be acknowledged though that some patients, such as the very elderly and seriously ill, are unlikely to realise the full long-term benefit of tight glycaemic control especially when associated with elevated risk of hypoglycaemia. There have also been publications questioning the benefits of intensive glycaemic control and the consequences for cardiovascular outcomes, such as the findings from the ACCORD study [17]. The cause of the increased mortality with intensive versus standard therapy in this study have still to be elucidated but they may indicate that aggressive titration to very low HbA1c levels is not appropriate for all (or indeed any) patients. Until there is an adequate explanation and association between the mortality risk and level of glycaemic control we are unable to effectively model this outcome with adequate understanding.

An issue not accounted in this study is the findings of an observational study by Bowker *et al*. [34] who reported a significantly higher rate of cancer-related mortality in patients exposed to sulfonylureas and insulin compared with patients exposed to metformin. This issue has received considerable attention recently with the publication of a study by Hemkens *et al*. [35] who reported a positive association between cancer incidence (with diagnosis of malignant neoplasm defined as the primary endpoint) and insulin glargine. However, the results were derived from studies conducted over a relatively short period of time (5 years), potentially suggesting that insulin (glargine) may accelerate the rate of development of existing tumours rather than stimulating malignant transformation and the formation of new ones [36]. Despite this concern the FDA has stated that variations in patient characteristics across treatment groups could have driven any difference in risk and has not acknowledged any confirmed link or advised any discontinuation of treatment [37]. With no firm evidence to link the use of insulin (or any other diabetes therapy) to increased cancer risk this has not been addressed in this analysis.

There are other implications that have not been addressed by this modelling analysis in relation to the management of diabetes patients that deserve further investigation. Although not reported in the two studies by Calvert and colleagues [20,21] there is accumulating evidence that type 2 diabetes patients are also poorly managed in terms of blood pressure and cholesterol levels [38-40]. It is highly likely that further clinical benefits not captured in this analysis could be realised if these additional patient management strategies were adhered to. Also, the similarities and differences between the levels of glycaemic control and time to initiation of insulin in the UK and other settings are unknown. It is not possible perhaps to translate these results directly to other countries but it would be surprising indeed if similar trends were not observed elsewhere and hence comparable outcomes.

It is known that progression to insulin therapy is often resisted by both patients and physicians [41]. The frequently cited reasons are both physical and psychological, for example a fear of injections, an inability to comply with the regimen or a feeling of failure in managing the disease so that insulin is now required for effective treatment. Patients themselves are also frequently unaware of the chronic and progressive nature of their condition and often do not understand the need for changes in therapies [42]. Regardless of the reasons for it, the consequences of failing to maintain glycaemic control are severe and this study clearly demonstrates the potential mortality and morbidity that could be avoided if guidelines and targets are adhered to.

## Conclusion

The immediate versus delayed initiation of insulin is projected to lead to substantial improvements in clinical outcomes over patient lifetimes. Data from the UK have shown that patients may typically delay the initiation of insulin by around 8 years despite poor glycaemic control and as a consequence mean life expectancy is reduced by over 7 months for each patient.

## Competing interests

GG and WJV are current or former employees of IMS Health who have received consultancy fees from Eli Lilly and Company.

ES and CH are employees of Eli Lilly and Company.

## Authors' contributions

All authors have contributed to the manuscript. GG designed and performed the modelling analyses for the study and drafted the manuscript. ES, CH and WJV contributed to and critically appraised the manuscript. All co-authors have seen and approved the final version of the paper and have agreed to its submission for publication. No others have participated in the project who require recognition here.

## Pre-publication history

The pre-publication history for this paper can be accessed here:


